# Bilateral Inferior Shoulder Dislocation

**DOI:** 10.5811/westjem.2015.11.24270

**Published:** 2014-12-01

**Authors:** Erica Cacioppo, James R. Waymack

**Affiliations:** Southern Illinois University School of Medicine, Division of Emergency Medicine, Department of Surgery, Springfield, Illinois

A 42-year-old male with a history of multiple shoulder dislocations presented to the emergency department via emergency medical services with both arms locked above his head, stating that he had been jumped at a bar and had since been unable to move his arms. A single anteroposterior chest radiograph (Figure) demonstrates bilateral inferior shoulder dislocations. The humeral head (white arrow) is displaced from the glenoid (yellow arrow) on each side.

Inferior shoulder dislocations are the rarest of all glenohumeral joint dislocations, accounting for less than 1%; and bilateral inferior dislocations are therefore extremely infrequent. The classic presentation is the patient whose arm is locked above his head in a hyper-abducted fashion. The inferior dislocation must first be converted to an anterior dislocation by slowly adducting the arm. The anterior dislocation can then be reduced using routine maneuvers.

In our case the patient underwent reduction with propofol sedation and was placed in bilateral shoulder slings. He was observed overnight by the orthopedic service for ethanol metabolism and was safely discharged home the next day in bilateral arm slings.

## Figures and Tables

**Figure:**
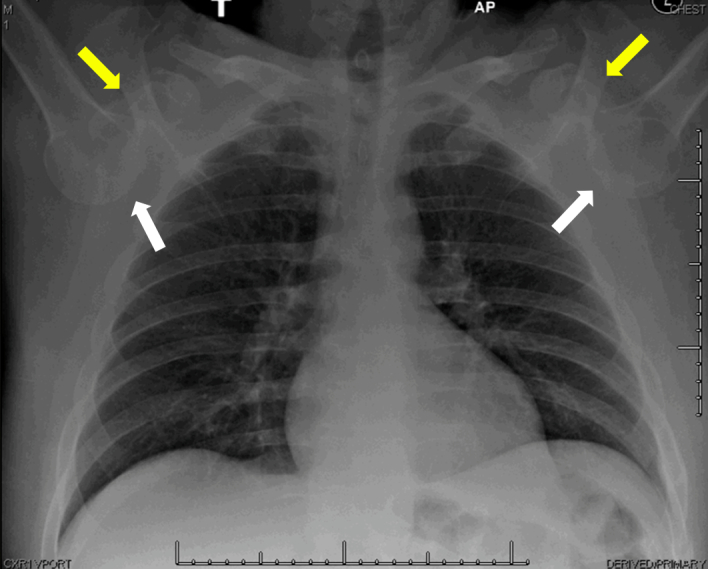
Radiograph of bilateral inferior shoulder dislocations. Humeral head (white arrow), displacement from glenoid (yellow arrow).

